# Supplementary material for: Integrative In Silico and In Vitro Transcriptomics Analysis Revealed Gene Expression Changes and Oncogenic Features of Normal Cholangiocytes after Chronic Alcohol Exposure
Source: Int J Mol Sci. 2019 Nov 28;20(23):5987. doi: 10.3390/ijms20235987 (PMC6928606; doi:10.3390/ijms20235987)
Supplement: Supplementary file 1 [file ijms-20-05987-s001.zip › ijms-635993-Supplementary for XML/Figure S1 Uncropped western blot intesity and ratio.pdf]

Figure S1. Detail information of western blot analysis

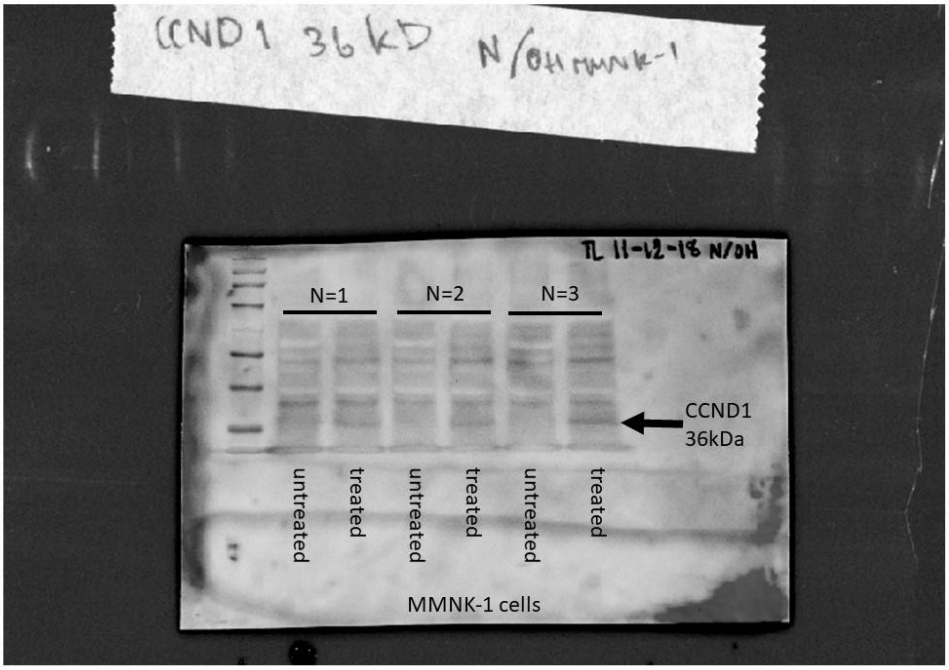

CCND-1 expression								
	N=1		N=2		N=3		Average	
	Untreated	Oh-treated	Untreated	Oh-treated	Untreated	Oh-treated	Untreated	Oh-treated
Density	402556	1213036	533808	948992	556428	1284272	497597.3333	1148766.667
ratio	1	3.013334791	1	1.777777778	1	2.308065015	1	2.308627056

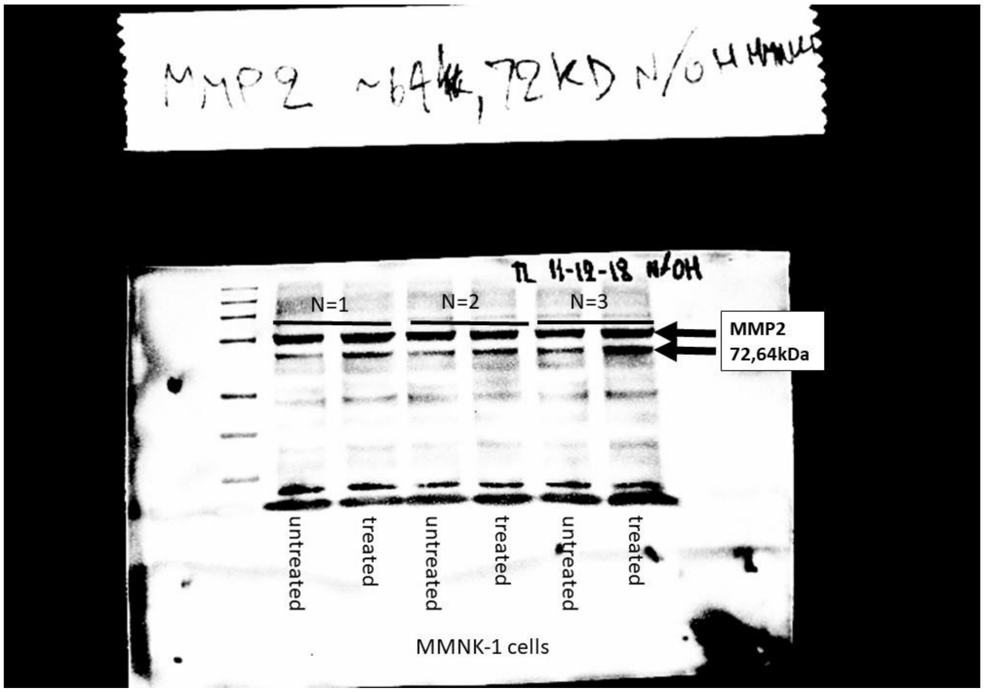

MMP-2 expression								
	N=1		N=2		N=3		Average	
	Untreated	Oh-treated	Untreated	Oh-treated	Untreated	Oh-treated	Untreated	Oh-treated
Density	1612864	1826000	1471096	1723084	1687224	3046516	1590394.7	2198533.33
ratio	1	1.132147534	1	1.171292696	1	1.80563814	1	1.38238223

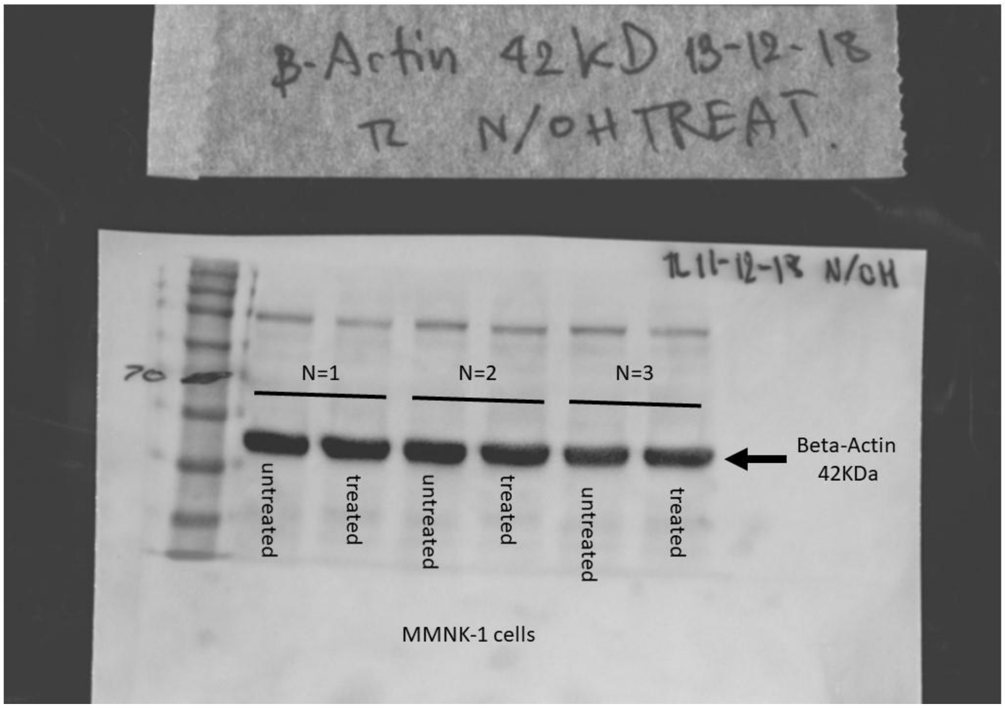
β-Actin 42KD 13-12-18
TL N/OH TREAT.
TL 11-12-18 N/OH
70
N=1
N=2
N=3
Beta-Actin
42KDa
untreated
treated
untreated
treated
untreated
treated
MMNK-1 cells
